# Venous thromboembolism prophylaxis practices in hematology/oncology patients admitted to neurological intensive care units

**DOI:** 10.3389/fcvm.2025.1573080

**Published:** 2025-07-30

**Authors:** Nada Alsuhebany, Lama Alfehaid, Danah Alodhaibi, Madhawi Mahdali, Sumaya Almohareb, Abdulrahman Alshaya

**Affiliations:** ^1^ Department of Pharmacy Practice, College of Pharmacy, King Saud bin Abdulaziz University for Health Sciences, Riyadh, Saudi Arabia; ^2^ Department of Pharmaceutical Care Services, King Abdulaziz Medical City, Ministry of National Guard-Health Affairs, Riyadh, Saudi Arabia; ^3^ King Abdullah International Medical Research Center, Riyadh, Saudi Arabia

**Keywords:** thrombosis, venous thromboembolism, hematology, oncology, neurological, anticoagulant activity

## Abstract

**Objective:**

Venous thromboembolism (VTE) presents a significant challenge for neurocritically ill patients with cancer due to the combined risks of thrombosis and bleeding. This study aimed to describe VTE prophylaxis practices among this high-risk population.

**Methods:**

This is a retrospective cohort study at a tertiary teaching hospital. Data were obtained for all patients admitted with neurocritical illness with a history of either solid tumors or hematological malignancies. The main outcome was the incidence of bleeding events in the neurocritical care unit (NCCU) using the International Society on Thrombosis and Haemostasis (ISTH) criteria. Other secondary outcomes were the incidence of thrombotic events, NCCU length of stay, and in-hospital mortality.

**Results:**

Out of the 168 patients screened, 43 patients were included, of which 38 patients (88.3%) had solid tumors, and 5 patients (11.6%) had hematologic malignancies. The majority of patients (81.3%) received chemical VTE prophylaxis during hospitalization. The incidence of major bleeding events was reported in 8 patients (21%) with solid tumors and one patient (20%) with hematologic malignancies, with no cases of thrombosis during hospitalization. Compared to the literature, the incidence of major bleeding events in our study is lower than indicated by a previous report on high bleeding risks in similar patient populations. The median duration of hospital stay was five days in the NCCU and 17 days in the hospital, with a 30-day mortality rate of 14%.

**Conclusion:**

Our study highlights the complexity of managing VTE prophylaxis in neurocritically ill cancer patients, emphasizing the need for a careful risk-benefit assessment. The absence of thrombotic events suggests effectiveness; however, bleeding risks warrant caution. These findings underscore the importance of individualized care and highlight the need for further research to refine prophylaxis protocols, thereby ensuring both safety and efficacy in this high-risk group.

## 1 Introduction

Venous thromboembolism (VTE) remains a major contributor to morbidity and mortality among hospitalized patients, particularly those in intensive care units (ICUs) (1–3). Neurocritically ill patients are at elevated risk due to prolonged immobilization, neurological deficits, and frequent contraindications to pharmacological thromboprophylaxis (4, 5). Concurrently, malignancy, especially hematological cancers, exerts a potent prothrombotic influence through tumor-mediated mechanisms, systemic inflammation, and treatment-related vascular injury (6, 7). When these two high-risk conditions coexist, as in neurocritical care patients with a history of cancer, the risk of VTE and bleeding becomes significantly amplified, yet remains poorly characterized (8).

Despite established guidelines supporting VTE prophylaxis in hospitalized patients, data specific to neurocritically ill individuals with cancer are limited. This subpopulation poses unique challenges, where standard prophylactic regimens may be either underutilized due to concerns about bleeding or applied inappropriately without individualized risk stratification. Previous epidemiologic studies have reported suboptimal prophylaxis rates in critical care, with as few as 40%–55% of at-risk patients receiving adequate VTE prevention (9–11). Moreover, cancer-related VTE carries a disproportionately high burden, with one-year survival rates as low as 47% (2), further emphasizing the need for early and appropriate preventive strategies.

In alignment with the mission of advancing VTE prevention in high-risk populations, this study aims to describe real-world patterns of VTE prophylaxis among neurocritically ill patients with a history of cancer. By highlighting current practice gaps, our findings seek to support the development of evidence-based strategies tailored to this vulnerable group, ultimately improving thromboprophylaxis efficacy and patient outcomes in neurocritical care.

## 2 Materials and methods

### 2.2 Study site and design

This was a retrospective cohort study conducted at a tertiary academic hospital affiliated with the Ministry of National Guard Health Affairs in Riyadh, Saudi Arabia. The hospital provides dedicated, highly specialized care for oncology and hematology patients. This study was approved by the Institutional Review Board at King Abdullah International Medical Research Center (SP20/446/R).

### 2.2 Study participants and treatment

All data were obtained from electronic medical records of adult hematology/oncology patients admitted with neurocritical illness between January 2016 and December 2023. Patients were excluded if they did not receive any VTE prophylaxis during their admission or were younger than 18 years old. VTE prophylaxis practices included pharmacological methods, primarily through the administration of subcutaneous unfractionated heparin (UFH) or low-molecular-weight heparin (LMWH), as well as mechanical methods involving pneumatic compression. The decision between pharmacological and mechanical prophylaxis was primarily determined by the medical team, based on their evaluation of the patients’ bleeding risk.

### 2.3 Study outcomes

The primary outcome was the incidence of bleeding events during the hospital stay in the neurocritical care unit (NCCU), as defined by the International Society on Thrombosis and Haemostasis (ISTH) criteria. Other secondary outcomes were the incidence of VTE events, including deep vein thrombosis (DVT) and pulmonary embolism (PE), NCCU length of stay, and in-hospital mortality. Based on ISTH, bleeding was defined as clinically overt bleeding associated with a decrease in hemoglobin (Hgb) level of 2 g/dl or more, requiring the transfusion of two or more units of packed red blood cells (RBCs), or occurring in a critical site (e.g., intracranial, intraspinal, intraocular, retroperitoneal, intra-articular, pericardial, or intramuscular with compartment syndrome). In addition to the demographic and clinical characteristics collected, the IMPROVE Score was computed at baseline to assess the risk of VTE in included patients.

### 2.4 Statistical analysis

Continuous data are presented as medians and interquartile ranges (IQRs), while categorical data are shown as counts and percentages. We used the Chi-square test or Fisher's exact test for comparing categorical outcomes. Statistical significance was set at 0.05. Data management and analysis were conducted using SPSS (version 30). All data were carefully reviewed to ensure completeness. In cases of missing data, efforts were made to retrieve the information from original sources. If retrieval was not possible, the missing data were excluded from the analysis. Exclusions were based on predefined criteria, and all decisions were documented to maintain transparency.

## 3 Results

**Figure 1:**
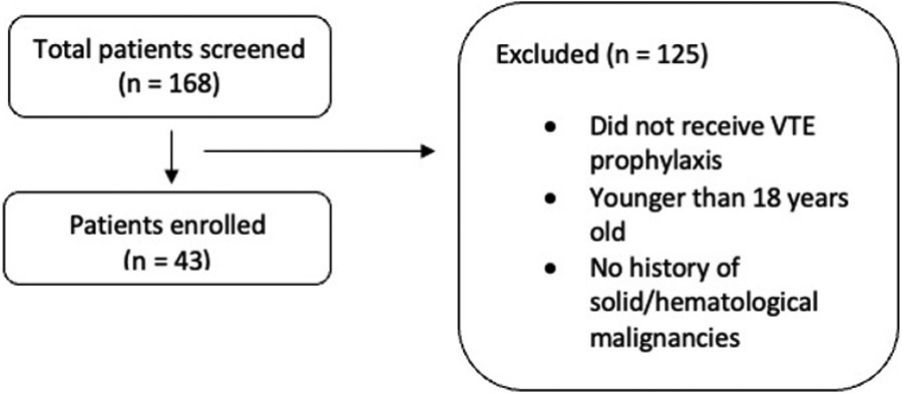
Flowsheet of the study screening and inclusion. Flowchart of patient selection for the study. A total of 168 patients were screened, with 43 patients enrolled. Exclusions (*n* = 125) were due to not receiving VTE prophylaxis, being younger than 18 years old, or having no history of solid or hematologic.

A total of 168 patients were screened in this retrospective study, and 43 participants were included (Figure 1).

### 3.1 Baseline characteristics

Among the 43 patients, 88.3% had solid tumor malignancies, while 11.6% had hematologic malignancies. The median age was 66 years (IQR 55.5–74), ranging from 19 to 91 years. The median body mass index (BMI) was 27.1 (IQR 23.1–30), and the median creatinine clearance (CrCl) was 70.9 ml/min (IQR 33.8–94). The median IMPROVE score was 3 (IQR 2–5). Primary or metastatic brain tumors were present in 30.2%. Home use of antiplatelets and anticoagulants was reported by 15.7% and 26.3% of patients, respectively. Regarding comorbidities, 18.6% had a history of ischemic stroke, and 7% had a history of VTE. The baseline platelet level was 270 × 10^9^/L (IQR 163–315). Additional baseline characteristics are detailed in (Table 1).

**Table 1:** Baseline characteristics.

Characteristics	Solid tumor malignancies *N* = 38	Hematologic malignancies *N* = 5	Total *N* = 43
Age, years			
Range	26–91	19–90	19–91
Median (IQR)	68 (57–74)	59 (35–62)	66 (55.5–74)
Gender, *n* (%)			
Male	20 (52.6)	4 (80)	24 (55.8)
BMI in kg/m^2^, median (IQR)	26.8 (23.4–30.1)	27.3 (23.1–27.4)	27.1 (23.1–30)
eGFR, median (IQR)	71.9 (39.2–93.4)	27.5 (17–94)	70.9 (33.8–94)
Chronic Kideny Disease (CKD)	25	5	30
CKD stage 1(GFR ≥90 ml/min/1.73 m^2^)	8	2	
CKD stage 2 (GFR 60–89 ml/min/1.73 m^2^)	9	0	
CKD stage 3 (GFR 30–59 ml/min/1.73 m^2^)	5	0	
CKD stage 4 (GFR 15–29 ml/min/1.73 m^2^)	2	3	
CKD stage 5 (GFR 15 ml/min/1.73 m^2^)	1	0	
PSH Neuro Absence^a^, *n* (%)	28 (73.6)	5 (100)	33 (76.7)
IMPROVE Score^b^, median (IQR)	4 (2–5)	2 (1–3)	3 (2–5)
HAS-BLED Score^c^, median (IQR)	1 (1–2)	2 (1–2)	1 (0–2)
Comorbidities, *n* (%)			
Ischemic stroke	7 (18.4)	1 (20)	8 (18.6)
History of renal dysfunction	6 (15.7)	1 (20)	7 (16.2)
History of liver dysfunction	5 (13.1)	0 (0)	5 (11.6)
History of VTE	3 (7.8)	1 (20)	4 (9.3)
Tumor diagnosis, *n* (%)			
Primary/metastatic brain tumors	13 (34.2)	0 (0)	13 (30.2)
Benign brain tumors	2 (5.2)	0 (0)	2 (4.6)
Others	23 (60.5)	5 (100)	28 (65.1)
Home medications, *n* (%)			
Antiplatelets	6 (15.7)	1 (20)	7 (16.2)
Anticoagulants	10 (26.3)	1 (20)	11 (25.5)
Baseline platelet level, median (IQR), 10^9^/L	273 (182–321)	53 (39–64)	270 (163–315)

^a^ PSH Neuro Absence means the absence of any paroxysmal sympathetic hyperactivity (PSH) neurological syndrome.

^b^ IMPROVE for VTE Risk Score predicts the 3-month risk of VTE in hospitalized patients.

^c^ HAS-BLED score is a clinical tool used to assess the risk of major bleeding in patients with atrial fibrillation.

### 3.2 NCCU admission interventions

The interventions during NCCU admission are summarized in (Table 2). Among the patients, 34.8% underwent major surgery. Anticoagulation therapy included UFH in 65.1% and LMWH in 44.1%, with 27.9% switching between these agents. Mechanical VTE prophylaxis was used in 18.6% without chemical prophylaxis. Antiplatelet therapy was continued in 23.2% of cases, while 46.5% experienced interruptions in VTE prophylaxis lasting over 24 h. Notably, 93.3% (14 out of 15) of patients with brain tumors received chemical VTE prophylaxis. Vasopressors were administered to 37.2% for a median duration of 2 days.

**Table 2:** NCCU admission conditions and interventions.

Endpoint	Solid tumor malignancies *N* = 38	Hematologic malignancies *N* = 5	Total *N* = 43
Major surgical procedures during admission, *n* (%)	13 (34.2)	2 (40)	15 (34.8)
Pharmacological VTE prophylaxis, *n* (%)	33 (86.8)	2 (40)	35 (81.3)
UFH	26 (68.4)	2 (40)	28 (65.1)
LMWH	18 (47.3)	1 (20)	19 (44.1)
Switched VTE prophylaxis	11 (26.3)	1 (20)	12 (27.9)
Mechanical VTE prophylaxis, *n* (%)	5 (13.1)	3 (60)	8 (18.6)
Inhospital antiplatelet use, *n* (%)	10 (26.3)	0	10 (23.2)
Inhospital VTE prophylaxis Interruption >24 h, *n* (%)	19 (50)	1 (20)	20 (46.5)
On vasopressors, *n* (%)	14 (36.8)	2 (40)	16 (37.2)
Duration of vasopressors, median, days	2	7	2
Worsening of platelet levels during hospitalization, *n* (%)	18 (47.3)	5 (100)	23 (53.4)
Platelet level at discharge, median (IQR), 10^9^/L	260 (151–360)	138 (60–141)	243 (138–345)

### 3.3 Major outcome results

**Figure 2:**
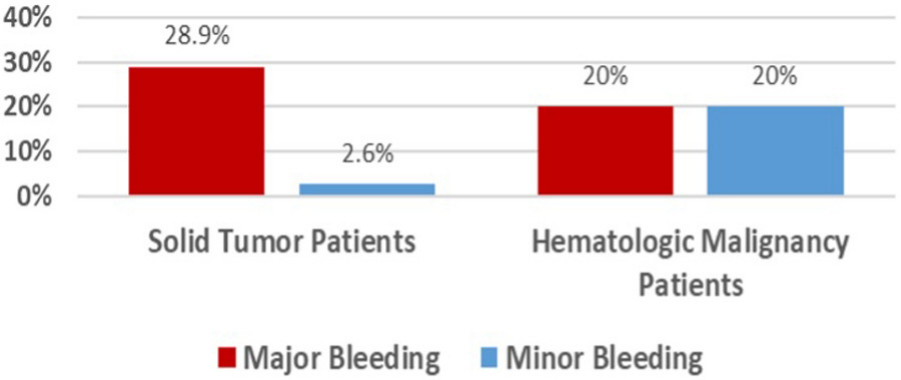
Incidence of bleeding.

Major bleeding events occurred in 20.9%, with 21% in those with solid tumors and 20% in hematologic malignancies (Table 3). Minor bleeding events were reported in one patient from each group (Figure 2). No cases of DVT or PE were observed during hospitalization. The median lengths of stay in the NCCU and the hospital were 5 days (IQR, 2–12) and 17 days (IQR, 10–62), respectively. The 30-day mortality rate was 13.9%, with 10.5% in solid tumor patients and 40% in hematologic malignancy patients. Among those with solid tumors experiencing major bleeding, 50% had brain tumors.

**Table 3:** Major outcome results.

Endpoint	Solid tumor malignancies *N* = 38	Hematologic malignancies *N* = 5	Total *N* = 43
Incidence of major bleeding events, *n* (%)	8 (21)	1 (20)	9 (20.9)
Intracranial hemorrahage	7 (18.4)	1 (20)	8 (18.6)
Incidence of minor bleeding events, *n* (%)	1 (2.6)	1 (20)	2 (4.6)
Incidence of VTE during hospitalization, *n* (%)	0 (0)	0 (0)	0 (0)
NCCU length of stay, median in days (IQR)	4 (1.25–17.75)	5 (5–8.5)	5 (2–12)
Hospital length of stay, median in days (IQR)	23.5 (11.75–60.5)	9 (7.5–35.5)	17 (10–62)
30-day mortality, *n* (%)	4 (10.5)	2 (40)	6 (13.9)

We compared UFH and LMWH in hematology/oncology patients (Table 4). There was no significant difference in cancer diagnosis distribution between the UFH and LMWH groups (*p* = 0.5601). Usage of UFH and LMWH was similar among patients with solid and hematologic malignancies. The incidence of major bleeding was comparable between the UFH (20.8%) and LMWH (18.1%) groups (*p* = 1.00). Minor bleeding occurred in 8.3% of the UFH group, with no cases in the LMWH group. No cases of VTE were reported in either group. The median NCCU stay was shorter for LMWH patients (3 days; IQR, 1.5–14.5 days) compared to UFH patients (10 days; IQR, 4–20 days). Hospital stays were comparable, with both groups having a median of 30 days. The 30-day mortality rate was higher in the UFH group (12.5%) compared to no mortality in the LMWH group.

**Table 4:** Major outcome results based on chemical VTE prophylaxis.

Endpoint	Unfractionated heparin *N* = 24	Low-molecular-weight heparin *N* = 11	*p*-value
Cancer diagnosis			
Solid tumor malignancy, *n* (%)	23 (95.9)	10 (91)	0.5601*
Hematologic malignancy, *n* (%)	1 (4.1)	1 (9)	–
Inhospital antiplatelet use, *n* (%)	4 (16.6)	3 (27.2)	0.6524**
Incidence of major bleeding events, *n* (%)	5 (20.8)	2 (18.1)	1.00**
Incidence of minor bleeding events, *n* (%)	2 (8.3)	0 (0)	–
Incidence of VTE during hospitalization, *n* (%)	0 (0)	0 (0)	–
NCCU length of stay, median in days (IQR)	10 (4–20)	3 (1.5–14.5)	–
Hospital length of stay, median in days (IQR)	30 (11–70)	30 (17–71)	–
30-day mortality, *n* (%)	3 (12.5)	0 (0)	–

* Was analyzed using Chi Square.

** Was analyzed using Fisher Exact.

## 4 Discussion

Hematology and oncology patients are at significantly elevated risk of VTE, including DVT and PE, which are major contributors to morbidity and mortality in this population (1, 8). This risk is further compounded in neurocritical care settings due to the convergence of multiple predisposing factors such as immobilization, active malignancy, organ dysfunction, and critical illness (8, 10). Current evidence emphasizes the importance of balancing thromboprophylaxis with the risk of bleeding in these patients, particularly those with central nervous system involvement or hemodynamic instability (12, 13). Despite established guidelines, including those from the National Comprehensive Cancer Network (NCCN), real-world practices in this context remain heterogeneous and poorly characterized, underscoring the need for additional observational data (14).

In our retrospective study, we evaluated baseline characteristics, NCCU admission variables, and clinical outcomes in patients with solid tumors and hematologic malignancies who were admitted to the NCCU. The median patient age was 66 years, with a slight male predominance (55.8%) and a median BMI of 27.1 (IQR 23.1–30). Comorbidities such as chronic kidney disease (16.1%) and prior ischemic stroke (18.6%) were present, both recognized risk factors for VTE (12, 13). The majority of patients (77%) received chemical VTE prophylaxis; however, 45% experienced interruptions exceeding 24 h. Vasopressor use was noted in 38% of patients for a median of 2 days. The incidence of major bleeding events was 20.9%, while 30-day mortality reached 14%. No thrombotic events were observed during hospitalization. The median NCCU and hospital lengths of stay were 5 days (IQR 2–12) and 17 days (IQR 10–62), respectively. Among brain tumor patients (93.3% of whom received chemical prophylaxis), four experienced major bleeding events. No significant differences were noted in bleeding rates or VTE incidence between UFH and LMWH; however, a trend toward reduced 30-day mortality was observed with LMWH.

Our findings regarding bleeding risk were lower than those in a study by Russell et al. that investigated the risks of bleeding and thrombosis in ICU patients with hematological malignancies; the overall bleeding risk was significant, reaching as high as 58% (15). This may be due to the small sample size affecting the power of our study. Furthermore, the demographic and clinical profiles of older age, male sex, higher BMI, and comorbid stroke or CKD mirror known predictors of cancer-associated thrombosis (9, 10, 12). Additionally, our HAS-BLED score was not elevated, which could be because the clinical score is not validated in cancer patients and only validated for patients with atrial fibrillation. The frequent interruptions in VTE prophylaxis highlight the challenge of maintaining consistent anticoagulation in the setting of fluctuating hemodynamic and neurologic status. Notably, the absence of thrombotic events, despite the high-risk cohort, suggests that the prophylactic strategies used may have been effective. However, this result should be interpreted with caution due to the study's small sample size.

Importantly, the use of chemical prophylaxis in brain tumor patients warrants critical evaluation. Despite guideline-based cautions regarding anticoagulation in this subgroup (14), high rates of prophylaxis were observed, and major bleeding occurred in several cases. This underscores the delicate balance required in managing thrombosis and bleeding risks in neurooncology. Similarly, vasopressor use, particularly norepinephrine, may influence cerebral perfusion and bleeding risk. Prior studies have demonstrated its effect on cerebral hemodynamics and potential associations with ischemic and hemorrhagic events (16).

Regarding pharmacologic strategies, our study found no statistically significant difference in bleeding or VTE rates between UFH and LMWH. However, the observed trend toward improved 30-day survival with LMWH aligns with previous research indicating lower risks of PE, bleeding, and HIT in critically ill cancer patients (16). This trend suggests that LMWH may offer a favorable risk-benefit profile. However, the potential influence of selection bias and confounding factors must be considered. Patients who received LMWH may have had differing baseline characteristics or clinical stability, which could have impacted the observed outcomes. Moreover, the choice of anticoagulant may have been guided by the clinician's judgment based on the individual patient's conditions. Importantly, renal function plays a crucial role in selecting and dosing anticoagulants. At our institution, specialized clinical pharmacists at NCCU regularly monitor renal function and provide dose adjustment recommendations accordingly. Upon retrospective review, we verified that patients with CrCl <30 ml/min were prescribed either UFH or renally adjusted LMWH (30 mg once daily), while those with CrCl <15 ml/min were managed exclusively with UFH. This approach reflects compliance with institutional dosing protocols and underscores the importance of individualized decision-making in mitigating bleeding risks. Future research should aim to validate these findings in larger cohorts and further investigate the factors contributing to the differences in clinical outcomes, including mortality.

Our study has several strengths, including the inclusion of all hematology/oncology patients admitted to the NCCU and the provision of a comprehensive assessment of their baseline characteristics, admission conditions, and major outcomes. However, there are limitations to acknowledge. The retrospective design and relatively small sample size may affect the robustness of our conclusions. Specifically, the small sample of patients with hematologic malignancies limits the generalizability of our findings to this group. Additionally, limited documentation hinders our ability to accurately assess the frequency of major and minor bleeding incidents. Another important limitation is the potential for selection bias in the decision to initiate pharmacologic vs. mechanical prophylaxis, which was based on clinical judgment rather than standardized criteria. This may have influenced bleeding outcomes, particularly given that patients perceived to be at higher bleeding risk were more likely to receive mechanical prophylaxis. As a result, the observed low rate of thrombotic events may not fully reflect the efficacy of prophylaxis, but rather the careful patient selection. These factors highlight the need for cautious interpretation and further research through prospective studies designed to reduce confounding and support evidence-based prophylaxis strategies in this high-risk population.

## 5 Conclusion

Our study highlights the complexity of managing VTE prophylaxis in neurocritically ill patients with cancer, emphasizing the critical need for a careful risk-benefit assessment. The absence of thrombotic events suggests that current prophylaxis strategies may be effective, yet the observed bleeding risks warrant cautious application. These findings underscore the importance of individualized patient care and the need for prospective studies to guide clinical decision-making. Our findings should be interpreted with caution, given the retrospective nature of the study and potential selection bias in the use of pharmacologic vs. mechanical prophylaxis. Future research should aim to refine prophylaxis protocols, ensuring safety and efficacy in this high-risk group. By addressing these challenges, we can enhance outcomes and optimize care for neurocritical patients.

## Data Availability

The raw data supporting the conclusions of this article will be made available by the authors, without undue reservation.
